# Urinary NGAL in gastrointestinal diseases can be used as an indicator of early infection in addition to acute kidney injury marker

**DOI:** 10.1002/jgh3.70009

**Published:** 2024-07-29

**Authors:** Yuichi Kojima, Atsunori Tsuchiya, Masaki Mito, Yusuke Watanabe, Yuzo Kawata, Kentaro Tominaga, Shuji Terai

**Affiliations:** ^1^ Division of Gastroenterology and Hepatology, Graduate School of Medical and Dental Sciences Niigata University Niigata Japan

**Keywords:** acute kidney injury, cirrhosis, gastrointestinal diseases, prognostic indicator, urinary neutrophil gelatinase‐associated lipocalin

## Abstract

**Background and Aim:**

Neutrophil gelatinase‐associated lipocalin (NGAL) is characterized by increased expression before the rise in serum creatinine and has been used as a biomarker for the early prediction of acute kidney injury (AKI). However, there have been no comprehensive analyses of its significance in gastrointestinal diseases. This study aimed to analyze the usefulness of measuring urinary NGAL levels in patients with gastrointestinal diseases.

**Methods:**

This study included 171 patients with a wide range of gastrointestinal diseases. Urinary NGAL levels were measured in all patients within 24 h of admission and 72 h later.

**Results:**

Urinary NGAL levels were higher in patients with acute pancreatitis and acute cholangitis/cholecystitis than in those with other diseases. Although lower than in these diseases, urinary NGAL tends to be higher in inflammatory bowel diseases, such as ulcerative colitis and Crohn's disease, as well as in acute and chronic liver diseases, and is higher in liver cirrhosis as the Child–Pugh grade increases. Furthermore, we found that the group with higher urinary NGAL levels, which continued to increase over time, had worse hospital stays and prognosis.

**Conclusion:**

Urinary NGAL could be used as an indicator of infectious diseases rather than an indicator of AKI in inflammatory bowel diseases and cirrhosis, and could predict the prognosis of patients with gastrointestinal diseases.

## Graphical abstract

Urinary NGAL is a known marker of acute kidney injury. In the gastrointestinal field, urinary NGAL increases rapidly in acute cholangitis and acute pancreatitis. However, urinary NGAL is also found to rise in cirrhosis, reflecting infection as the disease progresses. Furthermore, in cases where urinary NGAL did not improve after hospitalization, it was suggested that this may have an impact on prognosis.

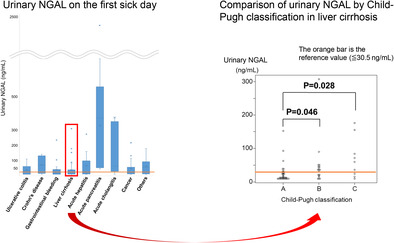

## Introduction

Neutrophil gelatinase‐associated lipocalin (NGAL) is a 24 kDa glycoprotein belonging to the lipocalin superfamily that is released from activated neutrophils during infection and inflammation.^1^ NGAL was isolated and identified in 2002 as a protein that induced the differentiation of undifferentiated renal progenitor cells into epithelial cells and nephrons.^2^ Because NGAL is characterized by its increased expression before the rise in serum creatinine, it has been used as a biomarker for the early prediction of acute kidney injury (AKI), a clinical syndrome that causes a rapid decline in renal function and renal tissue damage.^3^, ^4^, ^5^

Although several clinical studies have reported the prognostic value of urinary NGAL,^6^ it can be presumed that it can be elevated in many aspects of gastrointestinal diseases with infection and inflammation; however, there has been no comprehensive analysis of its overall significance.

This study aimed to capture the characteristics of the rise in urinary NGAL in each disease and find novels ways of utilizing urinary NGAL in patients with gastrointestinal diseases. We measured the levels in a wide range of gastrointestinal diseases, such as liver cirrhosis, gastrointestinal cancer, acute pancreatitis, acute cholangitis/cholecystitis, inflammatory bowel disease, and gastrointestinal bleeding. We first analyzed which aspects of these diseases are particularly prone to elevation. Additionally, further analysis of liver disease was conducted concerning liver function, inflammatory bowel diseases, and the prognostic significance of abnormal and unimproved NGAL levels in the field of gastroenterology, except for AKI.

## Methods

The study included 171 patients aged 20 years who were admitted to the Division of Gastroenterology and Hepatology, Niigata University Medical and Dental Hospital between April 2020 and March 2022 for liver cirrhosis, acute hepatitis, cancer, acute pancreatitis, acute cholangitis/cholecystitis, inflammatory bowel disease (ulcerative colitis and Crohn's disease), and gastrointestinal bleeding. Patients undergoing maintenance hemodialysis were excluded from the study. Age, sex, BMI, presence of chronic kidney disease, intensive care unit (ICU) or high care unit (HCU) admission rate, length of hospital stay, and mortality were retrospectively examined. Serum and urine samples were collected from all patients within 24 h of admission and 72 h later to measure serum creatinine, eGFR, white blood cell (WBC) count, neutrophil count, neutrophil/lymphocyte rate (NLR), CRP, and urinary NGAL. Urinary NGAL levels were measured in an outsourced laboratory (BML, Japan).

We comprehensively examined the association between urinary NGAL levels and gastrointestinal diseases. In cirrhosis, we investigated the association between urinary NGAL levels and Child–Pugh grade.^7^

Further, we investigated the association between urinary NGAL levels and prognosis of gastrointestinal diseases. We defined the group that fulfilled both of the following criteria: the rising high group and the group that did not fulfill both criteria as the control group and analyzed the prognosis of patients in both groups.

1. Urinary NGAL >30.5 ng/mL on Day 1.
2. Urinary NGAL on Day 3 > urinary NGAL on Day 1.

AKI was defined by one of the following^8^;

1. The serum creatinine level rises by more than 0.3 mg/dL within 48 h.
2. The serum creatinine level was known within the previous 7 days or increased by more than 1.5 times above the expected basal level.
3. Urine volume decreased by 0.5 mL/kg/h over a 6‐h period.

Chronic kidney disease was defined as one or both of the following conditions lasting for at least 3 months^9^:

1. eGFR<60 mL/min/1.73 m^2^.
2. Abnormal urinary findings such as proteinuria, a condition in which renal impairment is evident on imaging, blood tests, and pathological findings.

### Statistical analysis

Data are expressed as mean ± SD. EZR (Saitama Medical Center, Jichi Medical University, Saitama, Japan) was used for statistical analysis. Statistical significance was set at *P* < 0.05 by Kruskal–Wallis and Mann–Whitney *U* analyses. The correlation test used Spearman's rank correlation coefficient. Univariate and multivariate analyses were performed using multiple regression analysis with sex, age, BMI, presence of CKD, ICU/HCU admission rate, length of hospital stay, mortality, serum creatinine, eGFR, WBC count, neutrophil count, CRP, and urinary NGAL as covariates. Multivariate analysis was conducted using factors with *P* < 0.05 in the univariate analysis as covariates. Statistical significance was set at *P* < 0.05.

## Results

### Urinary NGAL was found to be associated with renal injury in gastrointestinal diseases, particularly in acute pancreatitis and acute cholangitis

**Figure 1:**
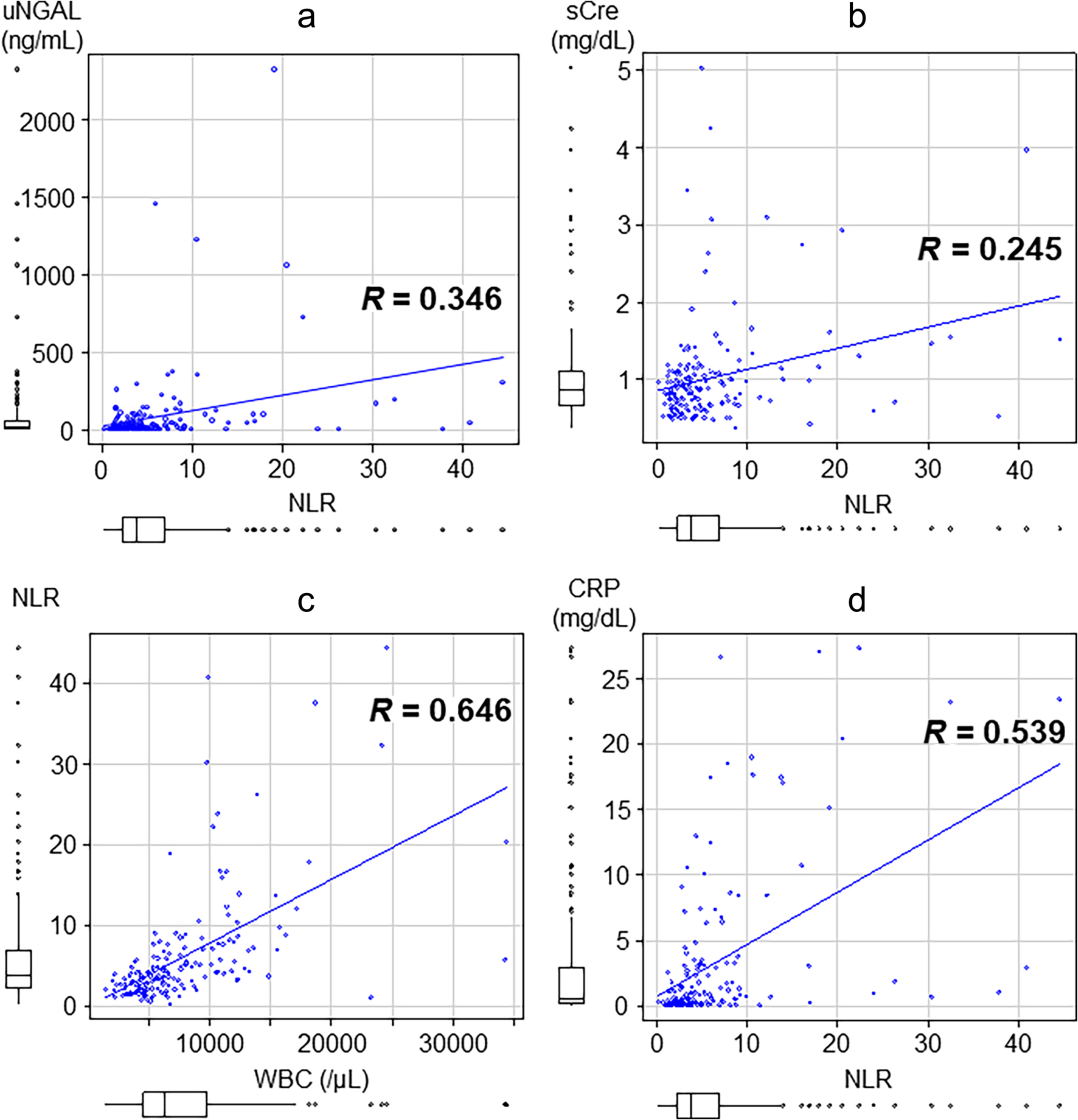
Correlation of NLR and urinary NGAL, blood test levels. (a) Correlation between NLR and urinary NGAL. (b) Correlation between NLR and serum creatinine. (c) Correlation between NLR and white blood cell. (d) Correlation between NLR and C‐reactive protein. CRP, C‐reactive protein; NLR, neutrophil/lymphocyte rate; sCre, serum creatinine; uNGAL, urinary neutrophil gelatinase‐associated lipocalin; WBC, white blood cell.

First, we collected and measured a wide range of urinary NGAL levels in patients hospitalized for gastrointestinal disease. The main diseases were inflammatory bowel diseases such as ulcerative colitis (13 cases), Crohn's disease (8 cases), gastrointestinal bleeding (16 cases), liver cirrhosis (61 cases), acute hepatitis (13 cases), acute pancreatitis (5 cases), acute cholangitis/cholecystitis (21 cases), and cancers such as pancreatic cancer, bile duct cancer, stomach cancer, and colorectal cancer (17 cases). The remaining 17 cases included ileus, ischemic enteritis, infected liver cysts, infected pancreatic cysts, cystic infection after acute pancreatitis, acute exacerbation of chronic pancreatitis, pancreatic abscess, Lemmel syndrome, biliary hemorrhage, autoimmune pancreatitis, splenic infarction, multiple small‐bowel ulcers, chronic diarrhea, dehydration, drug‐induced hypersensitivity syndrome (DIHS), and multiorgan failure (Table 1). Sex analysis included a population with a sex ratio of 110 (64.3%)/61 (35.7%) and an average age of 66.4 years (23–96 years) (Table 2). Urinary NGAL levels on the first and third days were 94.0 ng/mL (10–2330 ng/mL; reference value ≦30.5 ng/mL) and 58.2 ng/mL (10–723 ng/mL), respectively. The correlation between NLR and urinary NGAL and blood test levels was examined, and a weak correlation was found between NLR and urinary NGAL (*r* = 0.346) or creatinine (*r* = 0.245). Conversely, strong correlations were found between NLR and WBC (*r* = 0.646) or CRP (*r* = 0.539) (Fig. 1).

**Table 1:** Diseases of patients

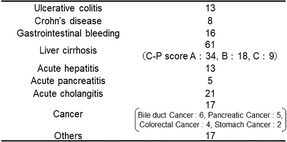

**Table 2:** Patient characteristics

Gender (males/females)	110 (64.3%)/61 (35.7%)
Age (years)	66.4 (23–96)
BMI (kg/m^2^)	24.0 (9.25–49.86)
CKD (+/−)	27 (15.8%)/144 (84.2%)
Serum Day 1	
CRP (mg/dL)	3.1 (0.01–27.35)
WBC (10^3^/μL)	7860 (1520–34 410)
Neutrophil (10^3^/μL)	5917 (890–31 657)
Creatinine (mg/dL)	1.03 (0.37–5.04)
eGFR (mL/min/1.73 m^2^)	66.60 (7.86–155.96)
Urine Day 1	
NGAL (ng/mL)	94.0 (10–2330)
Serum Day 3	
Creatinine (mg/dL)	0.96 (0.37–4.98)
eGFR (mL/min/1.73 m^2^)	67.12 (7.96–151.48)
Urine Day 3	
NGAL (ng/mL)	58.2 (10–723)
ICU or HCU stay (+/−)	27 (15.8%)/144 (84.2%)
Survival/transferred/death	160 (93.6%)/7 (4.1%)/4 (2.3%)
Hospital stay (days)	23.4 (3–190)

**Figure 2:**
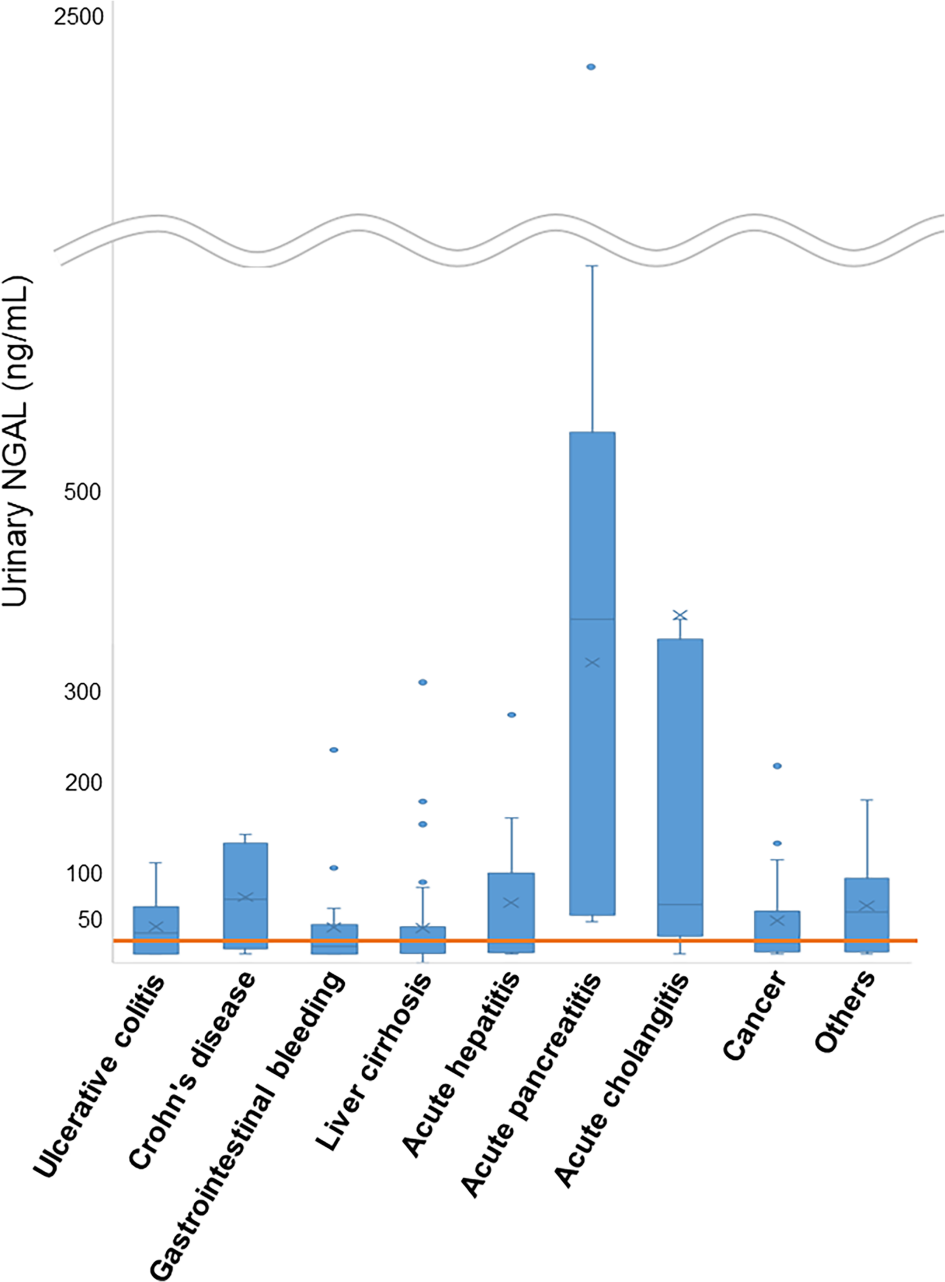
Urinary NGAL on the first sick day in all diseases. uNGAL on the first sick day was 39.7 ± 29.2 ng/mL for ulcerative colitis, 71.9 ± 54.7 ng/mL for Crohn's disease, 39.0 ± 56.0 ng/mL for gastrointestinal bleeding, 39.4 ± 50.2 ng/mL for liver cirrhosis, 66.0 ± 74.9 ng/mL for acute hepatitis, 317.2 ± 254.5 ng/mL for acute pancreatitis, 367.5 ± 608.2 ng/mL for acute cholangitis/cholecystitis, 46.3 ± 55.3 ng/mL for cancer, and 62.7 ± 51.6 ng/mL for others. Data are presented as the mean ± SEM. The orange bar is the reference value (≦30.5 ng/mL). uNGAL, urinary neutrophil gelatinase‐associated lipocalin.

**Figure 3:**
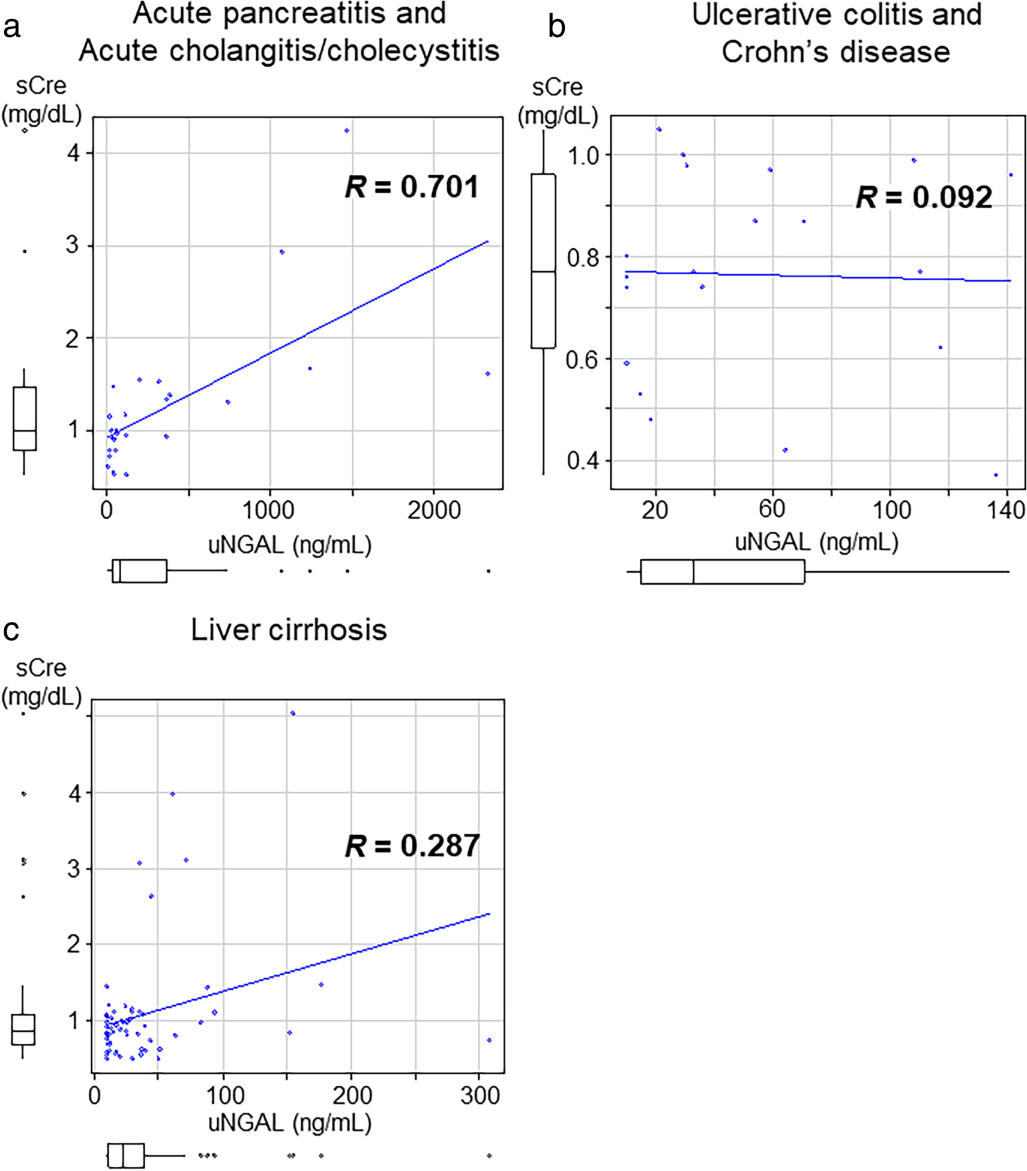
Correlation between urinary NGAL and serum creatinine. (a) Correlation between urinary NGAL and serum creatinine in acute pancreatitis and acute cholangitis/cholecystitis. (b) Correlation between urinary NGAL and serum creatinine in ulcerative colitis and Crohn's disease. (c) Correlation between urinary NGAL and serum creatinine in liver cirrhosis. sCre, serum creatinine; uNGAL, urinary neutrophil gelatinase‐associated lipocalin.

First, for all diseases, we analyzed the urinary NGAL levels on the first day of illness. The most prominent cases were acute pancreatitis (5 cases; 317.2 ± 254.5 ng/mL) and acute cholangitis/cholecystitis (21 cases; 367.5 ± 608.2 ng/mL) (Fig. 2). We analyzed the relationship between serum creatinine levels and the presence of AKI. Nineteen of the 26 patients (73.1%) had high urinary NGAL levels on the first sick day, 14 (53.8%) had high serum creatinine levels, and 11 (42.3%) had complications of AKI. In the present analysis, we found a correlation between urinary NGAL and serum creatinine (correlation coefficient: 0.701) (Fig. 3a) and considered that it could be an indicator of AKI, which has been conventionally described, but not necessarily for some diseases and could be used as another indicator. Therefore, further analysis was conducted.

### Urinary NGAL is also relatively high in hepatic disorders and Crohn's disease

We focused on diseases in which urinary NGAL levels were above normal on the first day, except for acute pancreatitis and acute cholangitis/cholecystitis. Urinary NGAL was elevated in ulcerative colitis (39.7 ± 29.2 ng/mL) and Crohn's disease (71.9 ± 54.7 ng/mL), especially in higher cases of Crohn's disease. High urinary NGAL levels were also observed in cases of liver cirrhosis (39.4 ± 50.2 ng/mL) and acute hepatitis (66.0 ± 74.9 ng/mL). Urinary NGAL levels on the first day of illness were higher than the normal range in 80 of 171 patients (46.8%), including 7 of 13 patients (53.8%) with ulcerative colitis and 4 of 8 patients (50%) with Crohn's disease. However, there were no cases of ulcerative colitis complicated by AKI and only one case of Crohn's disease. Thus, in ulcerative colitis and Crohn's disease, NGAL was not necessarily associated with AKI, suggesting an increase due to intestinal infection (Fig. 3b).

Because of the variability in liver cirrhosis, we decided to conduct a more in‐depth analysis of liver cirrhosis cases.

### In liver cirrhosis, the trend is higher as the disease progresses—a finding that may suggest the presence of infection

**Figure 4:**
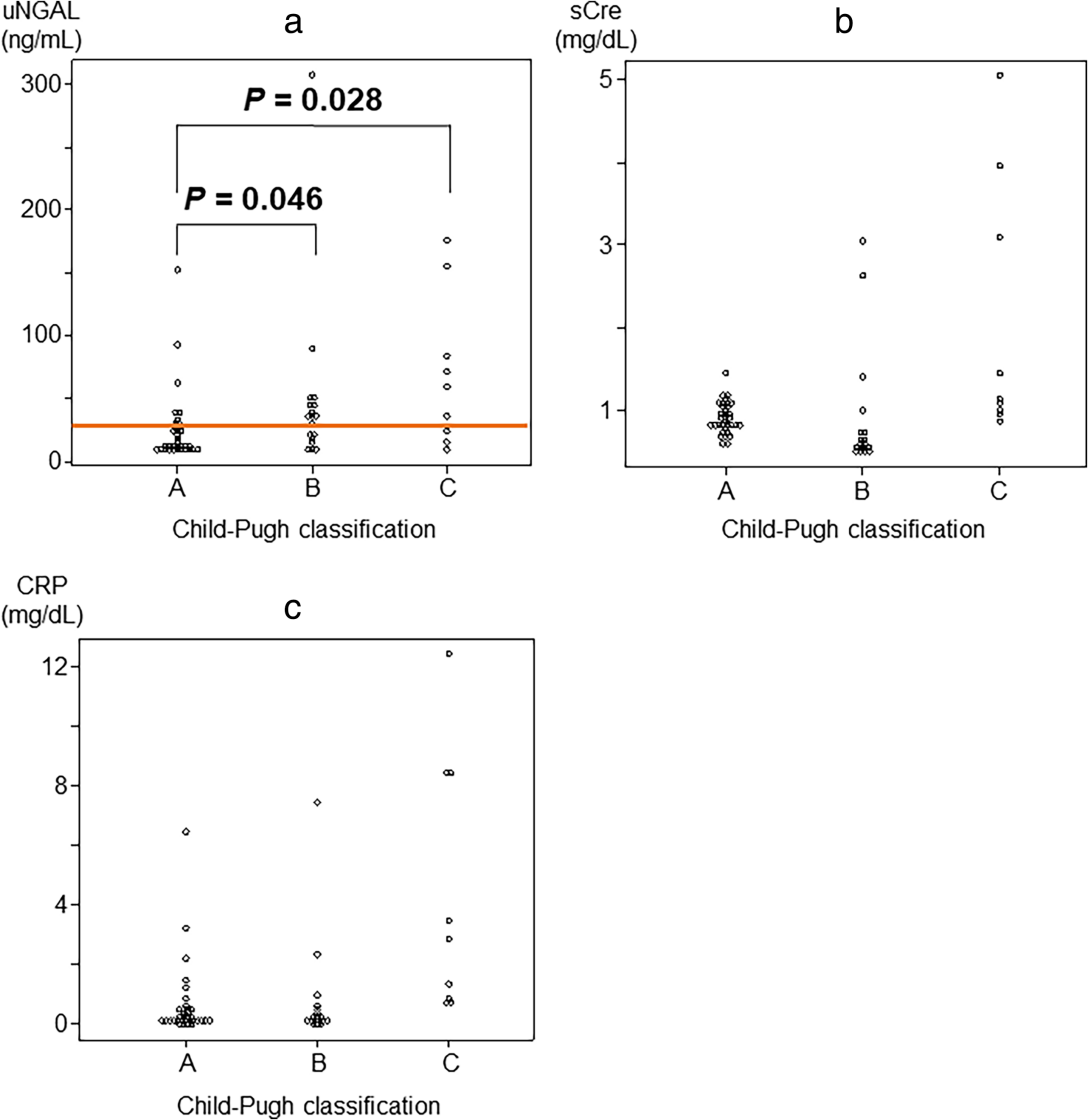
Comparison of urinary NGAL, serum creatinine, and CRP by Child–Pugh classification in liver cirrhosis. (a) Comparison of uNGAL by Child–Pugh classification. (b) Comparison of sCre by Child–Pugh classification. (c) Comparison of CRP by Child–Pugh classification. The orange bar is the reference value (≦30.5 ng/mL). CRP, C‐reactive protein; sCre, serum creatinine; uNGAL, urinary neutrophil gelatinase‐associated lipocalin. Mann–Whitney *U* analysis.

Data from the first sick day of patients with liver cirrhosis were analyzed using the Child–Pugh classification system as follows: Grade A: 34 cases 25.0 ± 28.0 ng/mL; Grade B: 18 cases 48.2 ± 65.8 ng/mL; Grade C: 9 cases 70.2 ± 56.7 ng/mL; as grade increased, urinary NGAL was noticeably higher (Fig. 4a). Urinary NGAL on the first sick day was high in 4 of 34 patients (11.8%) in Grade A, 10 of 18 patients (55.6%) in Grade B, and 6 of 9 patients (66.7%) in Grade C. Serum creatinine in the first sick day was Grade A: 34 cases 0.91 ± 0.19 mg/dL, Grade B: 18 cases 0.90 ± 0.73 mg/dL, Grade C: 9 cases 2.1 ± 1.5 mg/dL, showing no significant difference between Grade A and Grade B (Fig. 4b). In Grade A, there were no cases of AKI; however, four of nine cases (44.4%) of Grade C were associated with AKI. On the other hand, CRP was Grade A: 34 cases 0.63 ± 1.2 mg/dL; Grade B: 18 cases 0.77 ± 1.7 mg/dL; Grade C: 9 cases 4.4 ± 4.1 mg/dL, CRP tended to increase as the grade increased (Fig. 4c). Although urinary NGAL levels tended to increase as the Child–Pugh grade increased, there were many cases with abnormal values that were not necessarily associated with AKI (Fig. 3c).

This suggests that as liver cirrhosis progresses, there is inflammation in the body that gradually activates neutrophils and is associated with AKI, especially in Child–Pugh Grade C. However, in Grade B, urinary NGAL levels are high, but renal injury is scarce, possibly reflecting the presence of infection.

### Patients with urinary NGAL > 30.5 ng/mL on Day 1 and urinary NGAL on Day 3 > urinary NGAL on Day 1 tended to have a worse prognosis

We investigated whether urinary NGAL level could be a prognostic indicator of gastrointestinal diseases. We noticed that not only abnormal urinary NGAL on Day 1 but also patients who did not show improvement in urinary NGAL levels from Day 1 to Day 3 had a poor prognosis.

In univariate analysis, the rising high group had more women (66.7% *vs* 32.0%; *P* = 0.003), more chronic kidney disease (33.3% *vs* 13.7%; *P* = 0.031), higher serum creatinine on Day 3 (1.24 *vs* 0.93 mg/dL; *P* = 0.032), higher urinary NGAL on Day 3 (131.5 *vs* 49.6 ng/mL; *P* = 0.002), longer hospital stay (38 *vs* 22 days; *P* = 0.006), and higher mortality rate (16.7% *vs* 0.7%; *P* < 0.001). Multivariate analysis was performed using these factors as covariates, and sex (*P* = 0.020) and urinary NGAL level on Day 3 (*P* = 0.035) were identified as independent factors (Table 3).

**Table 3:** Univariate and multivariate analyses of prognostic indicators of gastrointestinal disease using urinary NGAL

	Satisfy ① and ② (*n* = 18)	Not satisfy ① and ② (*n* = 153)	Univariate analysis	Multivariate analysis	
			*P* value	Odds ratio (95% CI)	*P* value
Gender (% females)	12 (66.7%)	49 (32.0%)	**0.003**	0.249 (0.08–0.80)	**0.020**
Age (years)	70.4 (47–89)	66.0 (23–96)	0.251		
BMI (kg/m^2^)	24.5 (17.7–41.1)	24.0 (9.3–49.9)	0.716		
CKD (%)	6/18 (33.3%)	21/153 (13.7%)	**0.031**	3.67 (0.91–14.8)	0.068
Serum Day 1					
CRP (mg/dL)	2.67 (0.04–13.01)	3.16 (0.01–27.35)	0.737		
WBC (10^3^/μL)	7753 (3060–13 640)	7873 (1520–34 410)	0.926		
Neutrophil (10^3^/μL)	5752 (2070–11 180)	5936 (890–31 657)	0.879		
Creatinine (mg/dL)	1.30 (0.5–3.98)	1.00 (0.37–5.04)	0.071		
eGFR (mL/min/1.73 m^2^)	54.7 (10.84–116.91)	66.9 (7.86–155.96)	0.058		
Urine Day 1					
NGAL (ng/mL)	76.3 (32.2–363)	96.1 (10–2330)	0.752		
Serum Day 3					
Creatinine (mg/dL)	1.24 (0.51–4.1)	0.93 (0.37–4.98)	**0.032**	1.02 (0.40–2.62)	0.963
eGFR (mL/min/1.73 m^2^)	57.3 (23.87–104.15)	67.0 (7.96–151.48)	0.126		
Urine Day 3					
NGAL (ng/mL)	131.5 (39.5–548)	49.6 (10–723)	**0.002**	1.00 (1.00–1.01)	**0.035**
ICU or HCU stay (%)	5/18 (27.8%)	22/153 (14.4%)	0.142		
Hospital stay (days)	38 (7–190)	22 (3–126)	**0.006**	1.01 (0.99–1.03)	0.362
Death (%)	3/18 (16.7%)	1/153 (0.7%)	**<0.001**	15.6 (0.83–294)	0.066

①Urine Day 1 NGAL > 30.5 ng/mL. ②Urine Day 3 NGAL > Urine Day 1 NGAL. *P* < 0.05 was defined as a significant difference.

## Discussion

In this study, we analyzed the usefulness of urinary NGAL levels in a wide range of gastrointestinal diseases. Urinary NGAL levels tend to be higher in patients with acute pancreatitis and acute cholangitis/cholecystitis than in those with other diseases. Although lower than in these diseases, urinary NGAL tends to be higher in inflammatory bowel diseases, such as ulcerative colitis and Crohn's disease, as well as in acute and chronic liver diseases, and is higher in liver cirrhosis as the Child–Pugh grade increases. We considered that these could be used as indicators of infectious diseases rather than necessarily as indicators of AKI. We found that the group with higher urinary NGAL levels, which continued to increase over time, had worse hospital stay and prognosis.

Most studies on NGAL have focused on its relationship with AKI, a clinical syndrome that causes a sudden loss of renal function and renal tissue damage. Indeed, Mishra *et al*. reported the usefulness of NGAL as a marker for the early diagnosis of AKI after cardiac surgery.^10^ In gastrointestinal diseases, Pradeep Siddappa *et al*. reported the usefulness of NGAL as a marker to predict the severity of acute pancreatitis and AKI.^1^ AKI is often present in many severe phases of the disease and is often directly related to prognosis, so it is not surprising that NGAL is an important indicator. However, because NGAL is first released by activated neutrophils,^11^ we believe that it should be more closely examined as a marker reflecting the activated state of neutrophils and not be limited to AKI. In addition, NGAL is significantly elevated in acute pancreatitis and cholangitis without AKI. Min *et al*. reported the usefulness of NGAL as a marker to predict ICU admission rates and mortality due to pneumonia.^12^

In support of our data, Allegretti *et al*. reported the usefulness of NGAL as a prognostic marker for patients with end‐stage liver cirrhosis.^6^ Further, Gambino *et al*. reported that urinary NGAL is an independent predictor of in‐hospital mortality.^13^ We are particularly interested in the relationship between increased portal pressure and NGAL associated with progressive fibrosis in liver cirrhosis. In liver cirrhosis, severe dysbiosis is caused by various factors, including portal hypertension, submucosal edema, difficulty for immune cells to infiltrate, abnormal interepithelial tight junction (TJ) protein, disruption of the gut vascular barrier (GVB), and decreased farnesoid X receptor (FXR) signaling in the ileum, which also results in decreased barrier function and abnormal bacterial products and metabolites.^14^, ^15^ In patients with liver cirrhosis, the invasion of enteric bacteria and bacteria‐associated substances, such as lipopolysaccharide (LPS) and pathogen‐associated molecular patterns (PAMPs) (bacterial invasion is called bacterial translocation), can cause extremely serious liver and systemic conditions.^16^ These not only cause liver damage, but also lead to systemic inflammation. These conditions lead to cirrhosis‐associated immune dysfunction (CAID). The CAID is divided into two categories: low and high grade. It can be divided into low‐grade systemic inflammation, which is observed in patients with compensated cirrhosis without organ failure and some decompensated cirrhosis, and high‐grade systemic inflammation, which is observed in patients with acute‐on‐chronic liver failure (ACLF) and is associated with high rates of short‐term mortality. These immune abnormalities can easily lead to an infectious state.^17^, ^18^, ^19^ We presume that NGAL may capture latent infection and inflammation due to the invasion of such bacteria and their components, and AKI, as represented by hepatorenal syndrome, is a related symptom, although it may suggest prerequisite infection and inflammation. Similar to leaky gut in cirrhosis, many cases of inflammatory bowel disease causing intestinal barrier dysfunction also show elevated NGAL levels without renal dysfunction. Interestingly, Abdelsameea *et al*. showed that not only does urinary NGAL tend to be higher in cirrhosis, but it is also significantly higher in hepatocellular carcinoma. Although we did not search for hepatocellular carcinoma cases in this study, this is a very interesting report that also explores the mechanism behind the elevated levels.^20^ Oikonomou *et al*. reported that NGAL is elevated in active IBD and correlates with established inflammatory markers and disease activity. This finding strongly suggests that this marker may capture latent infection and inflammation in these diseases.^21^

Our study has some limitations. The patients receiving drugs that cause tubular necrosis, such as nonsteroidal anti‐inflammatory drugs (NSAIDs), were not excluded from this study. Also, this study shows that the number of cases per disease was reduced due to the analysis of a wide range of diseases. Despite these limitations, we believe that this study is significant because it identifies a new way to utilize NGAL in gastrointestinal diseases.

Despite these limitations, we have found new implications for urinary NGAL measurement in gastrointestinal diseases. Urinary NGAL may be used not only as an indicator of AKI but also as an indicator of infection in some diseases such as inflammatory bowel disease and liver cirrhosis. Furthermore, it has been suggested to be prognostic in a wide range of gastrointestinal diseases.

## Ethics approval

The study protocol was approved by the Committee of Niigata University (approval number: 2019‐0367).

## Data Availability

All data required to evaluate the conclusions of this study are provided in the main text of the manuscript. This study did not include data from external repositories. Additional data related to this study were obtained from the authors.
